# Analysis of the mechanism of curcumin against osteoarthritis using metabolomics and transcriptomics

**DOI:** 10.1007/s00210-023-02785-y

**Published:** 2023-11-08

**Authors:** Wenxiang Deng, Qinghu He, Wenan Zhang

**Affiliations:** 1 Department of Rehabilitation and Healthcare, Hunan University of Medicine, Huaihua, 418000 Hunan China; 2 College of Integrated Traditional Chinese and Western Medicine, Hunan University of Chinese Medicine, Changsha, 410208 Hunan China

**Keywords:** Curcumin, Osteoarthritis, UPLC-Q-TOF/MS, Transcriptomics, Metabolomics

## Abstract

Curcumin, a polyphenolic compound derived from the turmeric plant (*Curcuma longa*), has been extensively studied for its anti-inflammatory and anti-proliferative properties. The safety and efficacy of curcumin have been thoroughly validated. Nevertheless, the underlying mechanism for treating osteoarthritis remains ambiguous. This study aims to reveal the potential mechanism of curcumin in treating osteoarthritis by using metabolomics and transcriptomics. Firstly, we validated the effect of curcumin on inflammatory factors in human articular chondrocytes. Secondly, we explored the cellular metabolism mechanism of curcumin against osteoarthritis using cell metabolomics. Thirdly, we assessed the differences in gene expression of human articular chondrocytes through transcriptomics. Lastly, to evaluate the essential targets and elucidate the potential mechanism underlying the therapeutic effects of curcumin in osteoarthritis, we conducted a screening of the proteins within the shared pathway of metabolomics and transcriptomics. Our results demonstrated that curcumin significantly decreased the levels of inflammatory markers, such as IL-β, IL-6, and TNF-α, in human articular chondrocytes. Cell metabolomics identified 106 differential metabolites, including beta-aminopropionitrile, 3-amino-2-piperidone, pyrrole-2-carboxaldehyde, and various other components. The transcriptomic analysis yielded 1050 differential mRNAs. Enrichment analysis showed that the differential metabolites and mRNAs were significantly enriched in seven pathways, including glycine, serine, and threonine metabolism; pentose and glucuronate interconversions; glycerolipid metabolism; histidine metabolism; mucin-type o-glycan biosynthesis; inositol phosphate metabolism; and cysteine and methionine metabolism. A total of 23 key targets were identified to be involved in these pathways. We speculate that curcumin may alleviate osteoarthritis by targeting key proteins involved in glycine, serine, and threonine metabolism; inhibiting pyruvate production; and modulating glycolysis.

## Introduction

Osteoarthritis is widely recognized as a prevalent degenerative joint disease that significantly impacts the elderly population and is a primary contributor to disability worldwide. It affects approximately 200 million individuals globally (Liu et al., 2020). The prevalence of osteoarthritis among individuals aged 65 years and above can reach as high as 50%. Osteoarthritis has become a growing concern to doctors, patients, and society due to the aging population and increasing life expectancy. Joint pain is the most prominent symptom in patients with osteoarthritis and is often the main reason for seeking medical assistance (Carcolé et al., 2019). Furthermore, long-term chronic pain also negatively affects the psychological, spiritual, sleep, and social activities of patients (Miller et al., 2020; You et al., 2022), severely impacting the overall well-being of patients and imposing significant burdens on both individuals and society as a whole (Tajerian et al., 2018). Clinical and epidemiological studies have identified several factors associated with the development of osteoarthritis, including aging, obesity, joint instability, trauma, and joint inflammation (Chang et al., 2019). Furthermore, osteoarthritis is also affected by factors such as sex, genetics, and joint anatomy (Shen et al., 2019).

There are various pathological changes in the ligaments, muscles, cartilage, osteophytes, and synovial tissue of the affected joints, which may ultimately lead to the manifestation of symptoms such as joint deformity and dysfunction, and in severe cases, complete loss of joint function. Among these pathological changes, the deterioration of cartilage plays a significant role in the progression of osteoarthritis (Schulze-Tanzil, 2019). Cartilage is a white avascular and innervated tissue that covers the surface of joints (Bergholt et al., 2016). Cartilage plays a crucial role in dispersing joint bearing forces, cushioning impacts, and minimizing friction within and between joints (Rowland et al., 2018). Extracellular matrix (ECM) and chondrocytes are the main components of cartilage. ECM accounts for more than 90% of cartilage components. The primary constituents of ECM are proteoglycans and collagen fibers, which are essential for stress distribution in articular cartilage. Chondrocytes represent the sole cell type currently identified within cartilage. The deterioration of cartilage is firstly the change of the microenvironment of cartilage, and chondrocytes play an important role in maintaining the synthesis and metabolic balance of extracellular protein matrix within cartilage (Jeon et al., 2017). Currently, there are several treatment options for osteoarthritis, with pain relief and cartilage repair medication being the most frequently used approaches. The most commonly prescribed medications for pain associated with osteoarthritis are nonsteroidal anti-inflammatory drugs and opioid pain relievers. Several studies have highlighted safety concerns related to the long-term use of oral nonsteroidal anti-inflammatory drugs, such as hepatotoxicity, nephrotoxicity, increased risk of hypertension, and other cardiovascular events, as well as multiple adverse effects on the upper gastrointestinal tract (Iolascon et al., 2021; Lam et al., 2019; Wanchoo et al., 2017; Yoon et al., 2023; Frantz et al., 2018). Therefore, it is crucial to identify a pharmaceutical agent that offers improved safety and effectiveness in the treatment of osteoarthritis.

Turmeric (*Curcuma longa* L.), a traditional Chinese medicine, possesses anti-inflammatory properties attributed to its polyphenol curcuminoids (Panknin et al., 2023). Curcumin, a polyphenolic compound derived from the plant *Curcuma longa*, has been used for the treatment of many diseases, including cardiovascular disease, diabetes, and autoimmune diseases (Zhang & Zeng, 2019; Yaribeygi et al., 2023; Ataei et al., 2023; Kou et al., 2023). It has anti-inflammatory and anti-proliferative properties (Razali et al., 2022; Luo et al., 2023). Furthermore, it exhibits immunomodulatory effects on pathways responsible for regulating immune responses (Atabaki et al., 2020). The anti-osteoarthritis effect of curcumin has been reported in recent years. For example, both curcumin and tetrahydrocurcumin can prevent osteoarthritis symptoms and reduce the expression of pro-inflammatory cytokines in rats with estrogen deficiency (Zhang et al., 2023; Park et al., 2016). However, the underlying mechanism remains largely unclear.

Herein, we investigated the potential mechanism of curcumin in treating osteoarthritis by using metabolomics and transcriptomics. The potential targets and signaling pathways of curcumin in the treatment of osteoarthritis were identified and analyzed. This study offers novel insights and innovative approaches for conducting thorough investigations on the treatment of osteoarthritis.

## Materials and methods

### Culture and subculture of human articular chondrocytes

Human articular chondrocytes were obtained from Procell Life Science & Technology Co., Ltd. (Wuhan, China). The cells were cultured in the complete human articular chondrocyte complete culture medium (Procell) supplemented with 0.25% trypsin solution (Batch number: WH1622G051) and PBS buffer solution (Batch number: WH0022A071), both from Wuhan Puno Bio-Life Technology Co., Ltd. The culture was conducted in a 5% CO_2_, 20% O_2_, 37 °C incubator for 2 days. Subsequently, the medium was replaced, and the cells were subcultured at a cell confluence of 70–80%. When the confluence of the second passage chondrocytes reached 70–80%, the chondrocytes were digested with 0.25% trypsin (Procell Life Science &Technology Co., Ltd.), rinsed once with PBS, and centrifuged at 1500 ×*g* for 5 min. After re-suspension, the chondrocytes were seeded into 25 cm^2^ cell culture flasks, placed in the incubator for routine culture and passage, and regularly observed under an inverted microscope.

### Cell treatment and grouping

The chondrocytes of the second passage were divided into the blank group, the model group, the curcumin group, and the meloxicam group. The model group was intervened with 10 ng/mL of IL-1β (Multisciences (Lianke) Biotech, Co., Ltd., Hangzhou, China). The curcumin group was treated with 10 ng/mL of IL-1β and 10 μM of curcumin (MedChemexpress Biotechnology Inc., Princeton, NJ, USA). Cells in the meloxicam group received treatment with 10 ng/mL of IL-1β and 10 μM of meloxicam (MedChemexpress Biotechnology Inc.). Each group was intervened for 24 h, and 10 replicate wells were set up for each group. The cells in the blank group were cultured without any interventions.

### CCK-8 assay

Cell viability was detected with the CCK-8 kit (Elabscience Biotechnology Co., Ltd., Wuhan, China). Briefly, cells were seeded into a 96-well plate (5 × 10^3^/well). After culture for 24 h, cells were treated with curcumin (5 μM, 25 μM, 50 μM, 100 μM, and 200 μM) or meloxicam (5 μM, 10 μM, 20 μM, 100 μM, and 200 μM) for 24 h and 48 h. A blank control group was set up. Each group had 6 replicate wells. Following the intervention, 10 μL of CCK-8 solution was added to each well and incubated for 2 h. The optical density at 450 nm wavelength was then detected with a microplate reader.

### ELISA

The inflammatory factors IL-1β, IL-6, and TNF-α were measured using the Human IL-1β/IL-6/TNF-α ELISA kits (Shanghai Enzyme-linked Biotechnology Co., Ltd., China) following the kit instructions (Yoshizumi et al., 2016). The optical density at 450 nm wavelength was detected with a microplate reader.

### Metabolomics analysis

#### Metabolites extraction

The chondrocytes were mixed with 200 μL of water, followed by vortexing for 30 s. The samples were then subjected to three freeze-and-thaw cycles using liquid nitrogen. Next, the samples were sonicated for 10 min in an ice water bath. Protein was quantified using 50 μL of the homogenate. Subsequently, 600 μL of pre-cooled extract (− 40 °C) consisting of methanol and acetonitrile in a 1:1 ratio was added to the homogenized solution. After vortexing for 30 s, the samples were sonicated in an ice-water bath for 10 min, incubated at – 40 °C for 1 h, and then centrifuged at 24000 ×*g* at 4 °C for 15 min. A total of 700 μL of supernatant was transferred to EP tubes and dried in a vacuum concentrator. Subsequently, a solution consisting of methanol, acetonitrile, and water in a 2:2:1 ratio, supplemented with an isotopically labeled internal standard mixture, was introduced (Alseekh & Aharoni, 2021; Doppler et al., 2016). After vortexing for 30 s, the samples were sonicated for 10 min in an ice-water bath and centrifuged at 24000 ×*g* for 15 min at 4 °C. The resulting supernatant was transferred into a new glass bottle for LC/MS analysis. Quality control samples were prepared by combining aliquots of all sample supernatants.

#### LC-MS/MS analysis

LC-MS/MS analysis was performed using a UHPLC system (Vanquish, Thermo Fisher Scientific) equipped with a UPLC BEH Amide column (2.1 mm × 100 mm, 1.7 μm) and coupled with a Q Exactive HFX mass spectrometer (Orbitrap MS, Thermo) (Dunn et al., 2011; Want et al., 2010; Peng et al., 2015). The mobile phase consisted of 25 mmol/L ammonium acetate, 25 mmol/L aqueous ammonium hydroxide (pH = 9.75), and acetonitrile. The autosampler temperature was 4 °C, and the injection volume was 4 μL. The full scan MS/MS spectra were obtained in the Information Dependent Acquisition mode of the Q Exactive HFX mass spectrometer using the acquisition software (Xcalibur, Thermo). The ESI source conditions were sheath gas flow rate of 30 Arb, auxiliary gas flow rate of 25 Arb, capillary temperature of 350 °C, full MS resolution of 120,000, MS/MS resolution of 7500, collision energy of 10/30/60 NCE mode, spray voltage of 3.6 kV (positive) or − 3.2 kV (negative), respectively.

#### Data preprocessing and annotation

The raw data was converted to the mzXML format using ProteoWizard. Subsequently, an in-house program developed in R and based on XCMS (Smith et al., 2006) was used for peak detection, extraction, alignment, and integration. An in-house MS2 database (BiotreeDB) was then applied for metabolite annotations. The cutoff value for annotations was set at 0.3.

### RNA extraction, library construction, and sequencing

RNA was extracted and quantified by Shanghai Biotree Biomedical Technology Co., Ltd. An mRNA library was constructed, and sequence filtering and alignment were performed. The gene expression level was characterized using Stringtie and TMM, and FPKM values were calculated. The differential expression fold change (FC) was also calculated (Pertea et al., 2016; Kovaka et al., 2019; Pertea et al., 2015; Wang et al., 2020). Gene expression *Q* value was calculated using edgeR3 software. Differential genes were identified based on a *Q* value < 0.05 and FC > 2 or FC < 0.5 (Sahraeian et al., 2017; Robinson et al., 2010). Cluster analysis was performed using the Bioconductor package in R. Venn diagrams were generated by identifying common genes using Venn 2.1.0 (http://bioinformatics.psb.ugent.be/webtools/Venn/).

### Data analysis for metabolomics and transcriptomics

To reduce data dimensionality and visualize relationships of the metabolomics data, we utilized principal component analysis (PCA), a widely used technique for decreasing dataset dimensionality and revealing underlying structures (Abdi & Williams, 2010). For identifying differential metabolites, we conducted orthogonal partial least squares discriminant analysis (OPLS-DA), which incorporates orthogonal projections to latent structures and is suitable for handling multivariate and highly correlated data (Boccard & Rutledge, 2013). Metabolites with a VIP (Variable Importance in Projection) value greater than 1 and a p-value less than 0.05 (determined by the Student *t*-test) were considered to be significantly altered. Additionally, we utilized MetaboAnalyst 5.0, a powerful online metabolomics data analysis tool that offers a variety of analysis tools and statistical methods for preprocessing, exploratory data analysis, biological interpretation, and data visualization of high-throughput metabolomics data (Pang et al., 2021), to perform a joint analysis of metabolomics and transcriptomics.

### Statistical analysis

The statistical analysis for this study was conducted using SPSSAU (Statistical Product and Service Software Automatically) (Version 23.0), a web-based data analysis platform (spssau.com). The correlation between the two parameters was determined using the Spearman correlation. Wilcoxon tests were used to compare differences between two groups, while Kruskal-Wallis tests were employed for comparisons involving three or more groups. Statistically significant results were defined as bilateral *p*-values less than 0.05.

## Results

### Determination of the optimal concentrations of curcumin and meloxicam

**Fig. 1:**
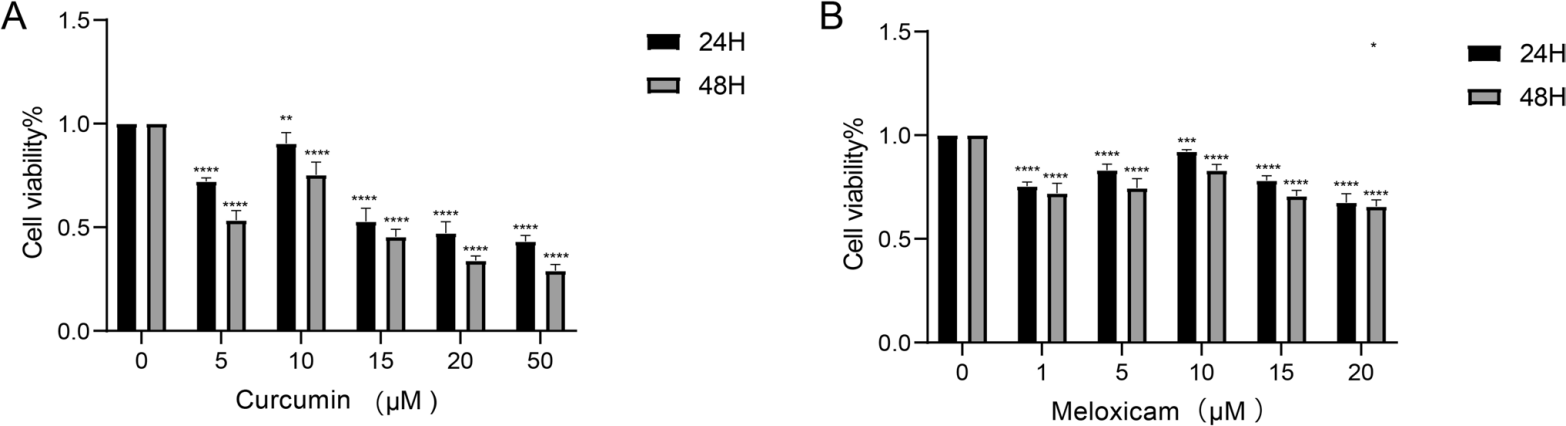
Effect of different concentrations of curcumin and meloxicam on cell viability of human articular chondrocytes. Cell viability was detected with CCK-8 assay. **A** Effect of curcumin on cell viability of human articular chondrocytes. **B** Effect of meloxicam on cell viability of human articular chondrocytes. Data are presented as mean ± SD. **p < 0.05, ***p < 0.001, ****p < 0.0001

Human articular chondrocytes were treated with different concentrations of curcumin (0 μM, 5 μM, 10 μM,15 μM, 20 μM, and 50 μM) and meloxicam (0 μM, 1 μM, 5 μM, 10 μM, 15 μM, and 20 μM) for 24 h and 48 h, respectively. Then, cell viability was detected with CCK-8 assay. The results showed that the cell viability of human articular chondrocytes was the highest when 10 μM of curcumin and meloxicam was used (Fig. 1A and B). Thus, both curcumin and meloxicam exhibited protective effects in human articular chondrocytes, with the concentration of 10 μM proving to be the most effective.

### Curcumin decreases the secretion of inflammatory cytokines in human articular chondrocytes

**Fig. 2:**
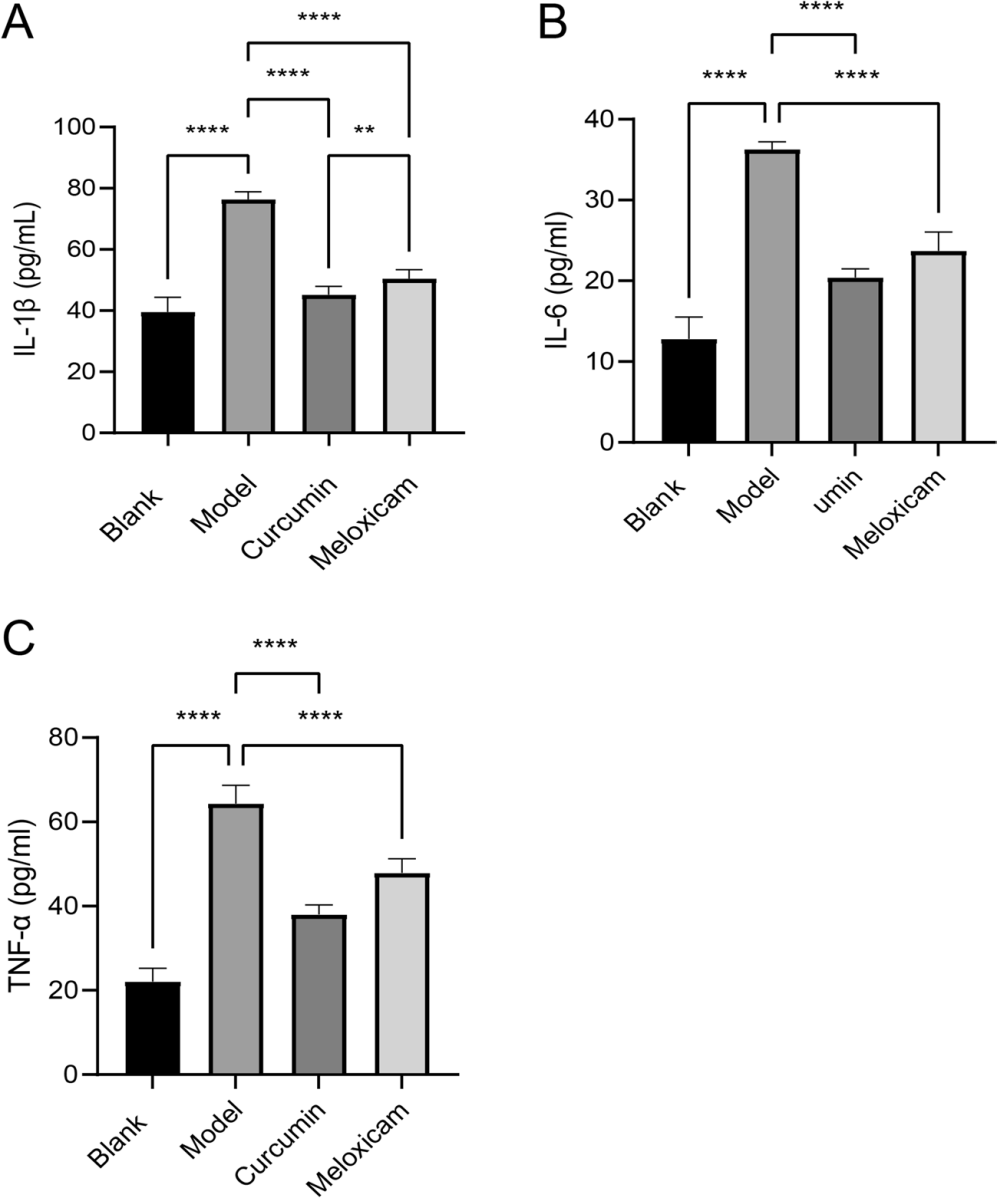
Effect of curcumin on cytokine levels. The cytokine levels were detected with ELISA. **A** IL-1β. **B** IL-6. **C** TNF-α. Data are presented as mean ± SD. **p < 0.05, ****p < 0.0001

In this study, to determine whether curcumin exerts anti-inflammatory effects, we measured the levels of IL-β, IL-6, and TNF-α in the culture supernatants of human articular chondrocytes by using ELISA. Compared with the model group, the concentrations of IL-1β (Fig. 2A), IL-6 (Fig. 2B), and TNF-α (Fig. 2C) in the blank group, curcumin group, and meloxicam group were significantly decreased (*p* < 0.05). In addition, the concentrations of IL-1β, IL-6, and TNF-α were significantly reduced in the curcumin group compared with the meloxicam group (*p* < 0.05). The results demonstrate that curcumin could reduce the secretion of inflammatory cytokines in human articular chondrocytes, exerting an anti-inflammatory effect.

### Changes in human chondrocyte metabolite profile in each group

**Fig. 3:**
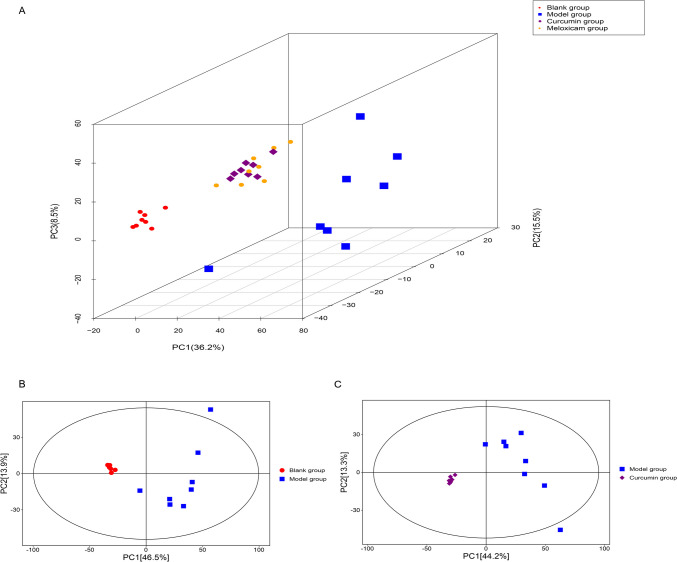
Changes of human chondrocyte metabolite profile in each group. **A** PCA chart of human articular chondrocyte metabolites in each group; the blank group, model group, curcumin group, and meloxicam group are located in different dimensions, indicating variations in the metabolite profiles of human articular chondrocytes among the four groups; **B** PCA scatter plot of the blank group and model group. The blank group and model groups are located in different dimensions, indicating differences in the metabolite profiles of human articular chondrocytes between the two groups; **C** PCA score scatter plot of the model group and curcumin group. The model and curcumin groups are located in different dimensions, suggesting differences in the metabolite profiles of human articular chondrocytes between the two groups. Note: The red circle represents the blank group, the blue rectangles represent model groups, the purple rhombus represents the curcumin group, and the yellow circle represents the meloxicam group

The profiles of human chondrocyte metabolites in each group were analyzed using PCA. As illustrated in Fig. 3A, the blank group, model group, curcumin group, and meloxicam group were located in distinct dimensions, suggesting that there are variations in the metabolite profiles of human articular chondrocytes among the four groups. Fig. 3B and C further demonstrate that the model group was differentiated from the blank group and the curcumin group, indicating significant differences in the metabolites of human chondrocytes across the various groups.

### Screening of differential metabolites in human chondrocytes of each group

**Fig. 4:**
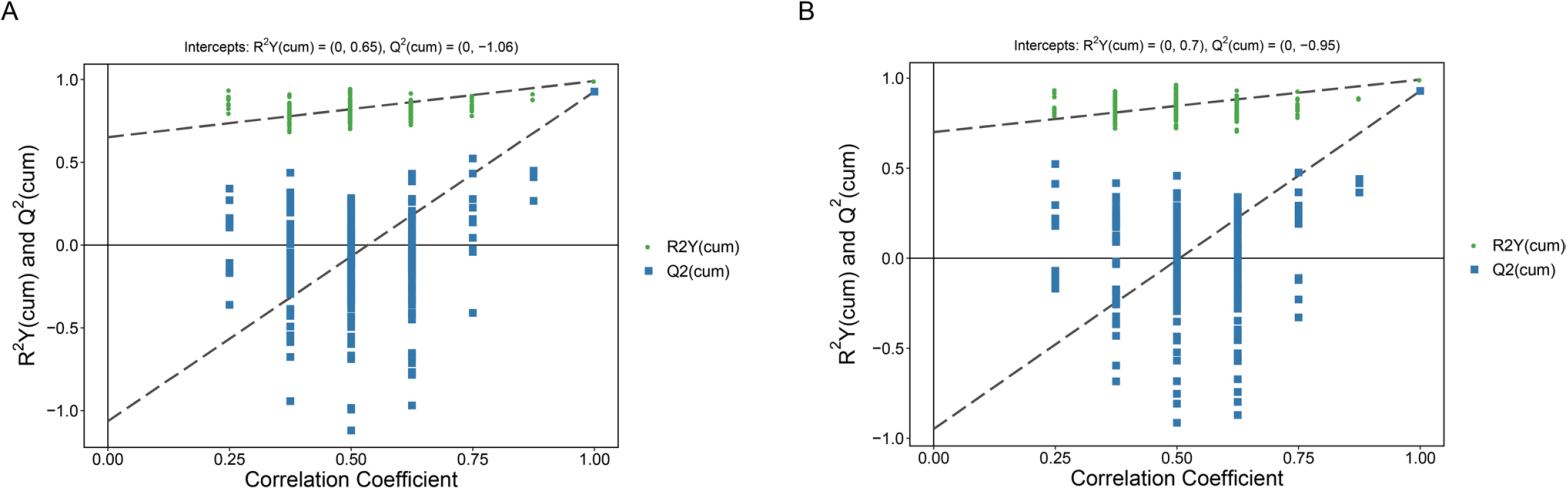
OPLS-DA model score charts. **A** Permutation test chart of the OPLS-DA model in the model group and the blank group; **B** permutation test chart of the OPLS-DA model in the model group and the curcumin group

**Fig. 5:**
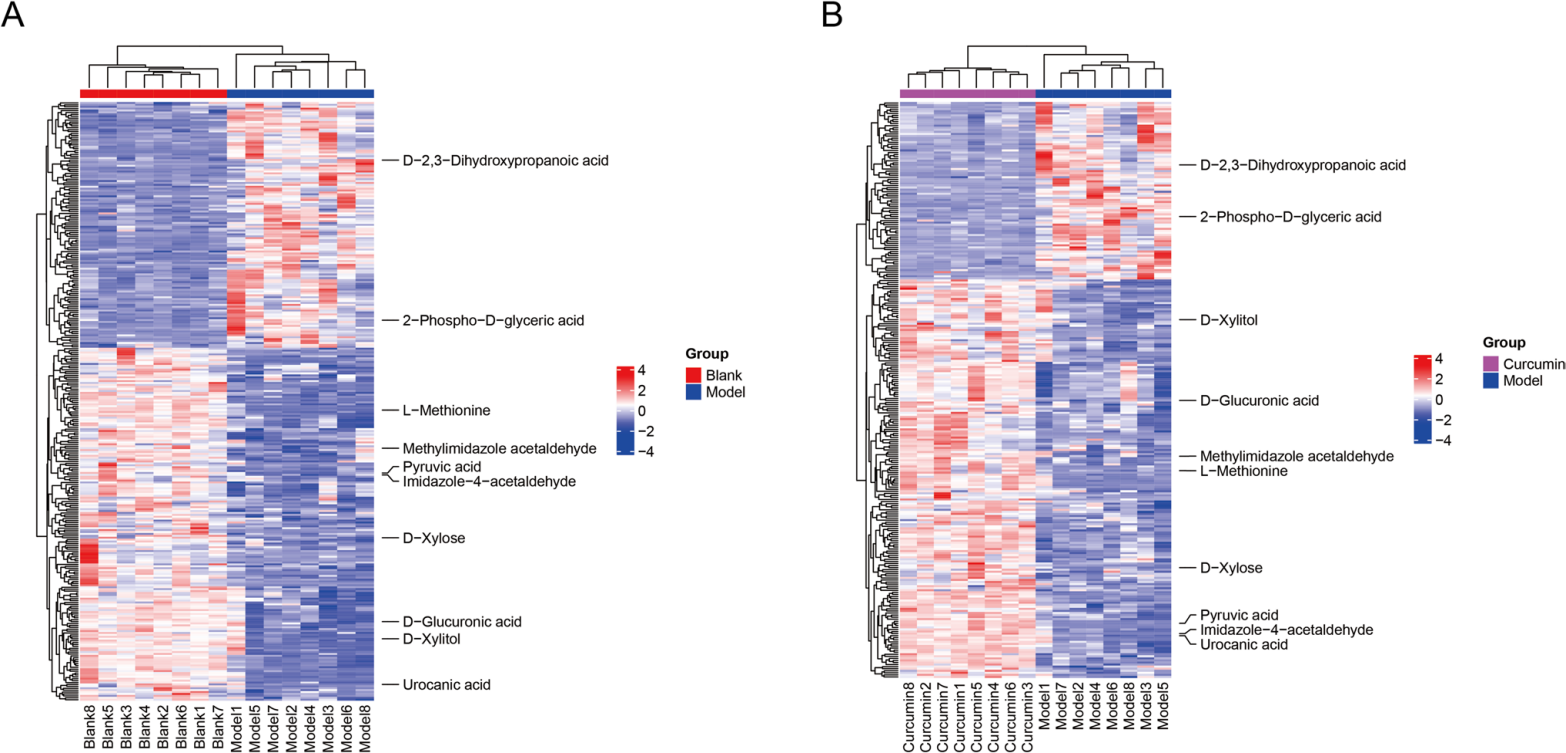
Heat map of differential metabolites. **A** Heat map of the top 10 differential metabolites between the blank group and the model group; **B** heat map of the top 10 differential metabolites between the model group and the curcumin group. Note: The abscissa represents the experimental group, and the ordinate represents the differential metabolites compared between the groups. The color blocks at different positions represent the relative expression of the metabolites at the corresponding positions. Red indicates high expression, and blue indicates low expression

To further identify the differential metabolites among the model group, the blank group, and the curcumin group, we performed OPLS-DA and conducted a permutation test. Metabolites with a VIP (Variable Importance in Projection) value greater than 1 and a *p*-value less than 0.05 (determined by the Student *t*-test) were regarded as significantly changed metabolites. As shown in Fig. 4, the model R2Y was very close to 1, and Q2 was relatively close to 1, indicating that the established model is in line with the real situation of the sample data, and the difference between the two groups of samples is significant. The hierarchical cluster analysis was conducted on differential metabolites, and a heat map was plotted. There were 314 differential metabolites between the blank group and the model group (Fig. 5A) and 279 differential metabolites between the model group and the curcumin group (Fig. 5B).

### Statistical analysis of differential metabolites

After identifying the common metabolites between the model group and the blank group, as well as between the model group and the curcumin group, statistical analysis was conducted using SPSSAU (Version 23.0) to determine the normalized peak areas of the metabolites. Finally, 106 significantly different metabolites were identified (Table 1).

**Table 1:** Summary of 106 differential metabolites of human articular chondrocytes

ID	Name	RT	M/Z	Blank vs model	Model vs curcumin
1	beta-Aminopropionitrile	6.11	71.06	↑**	↓**
2	3-Amino-2-piperidone	3.42	115.09	↑**	↓**
3	Pyrrole-2-carboxaldehyde	5.15	96.04	↑**	↓**
4	Pyruvic acid	2.76	87.01	↑**	↓**
5	Ethyl butyrate	1.12	115.08	↑**	↓**
6	Cholic acid	3.93	407.28	↑**	↓**
7	O-Phosphoethanolamine	7.9	140.01	↓**	↑*
8	N6-Methyladenosine	2.01	282.12	↑*	↓**
9	2-Phospho-d-glyceric acid	7.89	184.99	↓*	↑**
10	Thiamine	5.78	265.11	↑**	↓**
11	Phosphoenolpyruvic acid	7.89	166.97	↓**	↑**
12	7-Methylguanosine	3.47	298.11	↑**	↓**
13	l-Palmitoylcarnitine	3.26	400.34	↑**	↓*
14	d-Glutamine	6.09	145.06	↑*	↓**
15	Octadecylamine	2.29	270.32	↓**	↑**
16	l-Methionine	5	150.06	↑**	↓**
17	N-Acetyl-glucosamine 1-phosphate	7.7	300.05	↓*	↑*
18	l-Arginine	8.68	173.1	↑*	↓**
19	Phosphorylcholine	8.09	184.07	↓*	↑*
20	d-Xylose	2.74	149.04	↑**	↓**
21	2-Hydroxyethanesulfonate	2.85	124.99	↑**	↓**
22	Decanoylcarnitine	3.53	316.25	↑*	↓**
23	Urocanic acid	5.04	139.05	↑*	↓**
24	d-2,3-Dihydroxypropanoic acid	3.41	105.02	↓**	↑**
25	Uridine	2.72	243.06	↑**	↓**
26	N-Acetylhistidine	5.47	198.09	↑**	↓*
27	Quinone	5.13	109.03	↑**	↓**
28	Phenylalanylphenylalanine	2.85	313.16	↑**	↓**
29	Ricinoleic acid	0.85	297.24	↑**	↓*
30	Prostaglandin E2	1.77	351.22	↓**	↑**
31	l-Malic acid	7.8	133.01	↑**	↓**
32	d-Glucuronic acid	6.64	193.04	↑**	↓**
33	l-Nicotine	1.02	163.12	↓**	↑*
34	Pterolactam	5.34	116.07	↓**	↑*
35	Tetradecanoylcarnitine	3.33	372.31	↑*	↓**
36	l-trans-alpha-Amino-2-carboxycyclopropaneacetic acid	5.09	160.06	↑**	↓**
37	N2,N2-Dimethylguanosine	3.33	310.12	↑**	↓*
38	Trigonelline	4.94	138.05	↑**	↓**
39	Azelaic acid	3.48	187.1	↓*	↑*
40	l-Gulonic gamma-lactone	1.51	177.04	↑**	↓**
41	d-Xylitol	3.61	151.06	↑**	↓**
42	Pyrophosphate	8.52	176.94	↓**	↑**
43	N-Acetylaspartylglutamic acid	7.72	303.08	↑**	↓**
44	1,3-Diacetoxy-4,6,12-tetradecatriene-8,10-diyne	3.42	301.13	↑**	↓**
45	Suberic acid	3.46	173.08	↓**	↑**
46	Maleic acid	4.99	115	↑**	↓**
47	Beta-Alanine	6.25	90.06	↑**	↓**
48	l-Canavanine	0.37	221.09	↓*	↑*
49	15-Deoxy-d-12,14-PGJ2	1.76	315.2	↓**	↑**
50	3-Guanidinopropionic acid	2.88	132.08	↑*	↓**
51	Mannitol	4.93	181.07	↓**	↑**
52	Glutamylglutamic acid	7.84	275.09	↑**	↓**
53	Valyl-serine	5.24	205.13	↑*	↓*
54	Triethylamine	0.59	102.13	↑**	↓**
55	Mukonine	4.6	256.09	↑**	↓*
56	l-Prolyl-l-proline	7.05	213.12	↓**	↑**
57	Resveratrol	4.55	227.07	↓**	↑**
58	2,4-Diamino-6-nitrotoluene	3.46	168.08	↑*	↓**
59	Uridine diphosphate glucuronic acid	8.22	579.03	↓*	↑*
60	Pyrrole-2-carboxylic acid	8.28	110.02	↓**	↑**
61	Muramic acid	7.65	252.11	↓**	↑**
62	l-Proline	1.2	116.07	↓**	↑**
63	Gentiatibetine	3.64	166.09	↑**	↓*
64	Anonaine	6.99	266.12	↓**	↑**
65	Desaminotyrosine	0.09	165.05	↑**	↓**
66	Cimifugin	3.48	307.11	↑**	↓**
67	1-Methylhypoxanthine	2.38	151.06	↑**	↓**
68	2-Hydroxyfluorene	2.88	183.08	↑**	↓**
69	Imidazole-4-acetaldehyde	5.17	111.06	↑**	↓**
70	N-[(4-Hydroxy-3-methoxyphenyl)methyl]octanamide	3.49	280.19	↑**	↓**
71	N-Methyl-d-aspartic acid	5.61	146.05	↓*	↑*
72	Ascorbic acid	6.34	175.02	↑**	↓**
73	4-(2-Aminophenyl)-2,4-dioxobutanoic acid	6.81	208.06	↓**	↑**
74	LysoPE(18:1(9Z)/0:0)	3.61	478.29	↑**	↓*
75	l-Alanine	0.81	90.06	↓*	↑*
76	Stachyose	8.44	665.22	↓**	↑**
77	6-Methyladenine	1.91	150.08	↓*	↑*
78	beta-d-Glucosamine	6.24	180.09	↓**	↑**
79	Linatine	6.55	260.12	↑**	↓**
80	6-Hydroxy-1H-indole-3-acetamide	0.76	191.08	↑*	↓**
81	Koenimbine	8.73	294.15	↓**	↑**
82	Gluconolactone	2.76	177.04	↑**	↓**
83	Aminoadipic acid	6.23	162.08	↓**	↑**
84	Alpha-Hydroxyisobutyric acid	4.66	103.04	↑**	↓*
85	Hydroxykynurenine	6.81	225.09	↓**	↑**
86	Uracil	1.24	111.02	↓**	↑*
87	l-2,4-Diaminobutyric acid	9.34	119.08	↑**	↓**
88	Caffeine	5.98	195.08	↑**	↓**
89	Homocitrulline	7.15	190.12	↑**	↓*
90	Demethylated antipyrine	0.82	175.09	↑**	↓**
91	Epsilon-(gamma-glutamyl)-lysine	7.84	274.14	↑**	↓*
92	Frenolicin B	6.46	329.1	↓**	↑**
93	Methylimidazole acetaldehyde	2.92	125.07	↑**	↓**
94	Monomethyl glutaric acid	3.49	145.05	↓**	↑**
95	Glutaminylcysteine	6.16	250.09	↓**	↑**
96	Glutaric acid	1.21	131.03	↑*	↓*
97	Phloretin	5.01	273.07	↑*	↓**
98	Phytosphingosine	1.03	318.3	↓**	↑**
99	gamma-Glutamylleucine	6.26	261.14	↑**	↓**
100	L,L-Cyclo(leucylprolyl)	7.95	233.12	↓*	↑**
101	2-Fucosyllactose	6.64	511.17	↑**	↓**
102	Triacetin	8.69	219.08	↑*	↓**
103	Melibiose	6.37	381.07	↓*	↑**
104	Deoxyribose 1-phosphate	1.32	213.02	↑**	↓**
105	Glutaminylserine	6.19	234.11	↑**	↓**
106	1-Isothiocyanato-6-(methylsulfinyl)hexane	7.26	206.07	↓**	↑**

Note: *RT*, retention time; *M/Z*, mass charge ratio; ↑represents increase, ↓ represents decrease; *P < 0.05, **P < 0.01

### Analysis of differential mRNA in human articular chondrocytes

**Fig. 6:**
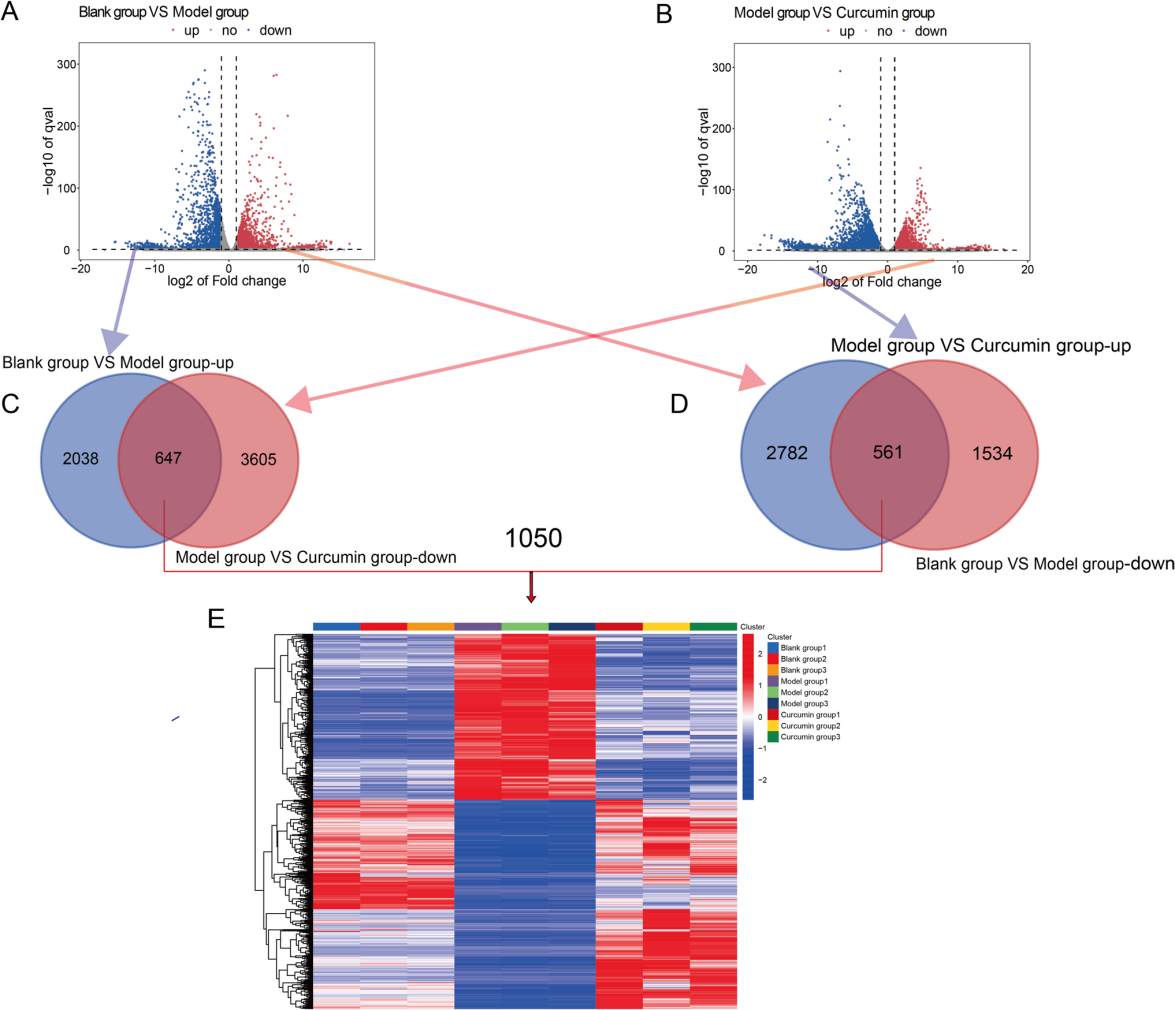
Differential mRNA analysis of human articular chondrocytes. **A** Differential mRNA volcano map of blank group and model group; **B** differential mRNA volcano map of model group and curcumin group; **C** differential mRNA Venn diagram of blank group and model group; **D** differential mRNA Venn diagram of the model group and curcumin group; **E** differential mRNA clustering heat map. The darker the red, the higher the expression, and the darker the green, the lower the expression

Compared with the blank group, the model group had a total of 4780 differentially expressed genes (DEGs), of which 2685 were upregulated and 2095 were downregulated. Compared with the model group, there were 7595 DEGs in the curcumin group, of which 3343 were upregulated and 4252 were downregulated. The cluster analysis of the differentially expressed genes (DEGs) in the three groups revealed a total of 1050 DEGs that were dysregulated in the model group and restored by curcumin, as illustrated in Fig. 6.

### Integrative analysis of metabolomics and transcriptomics

**Fig. 7:**
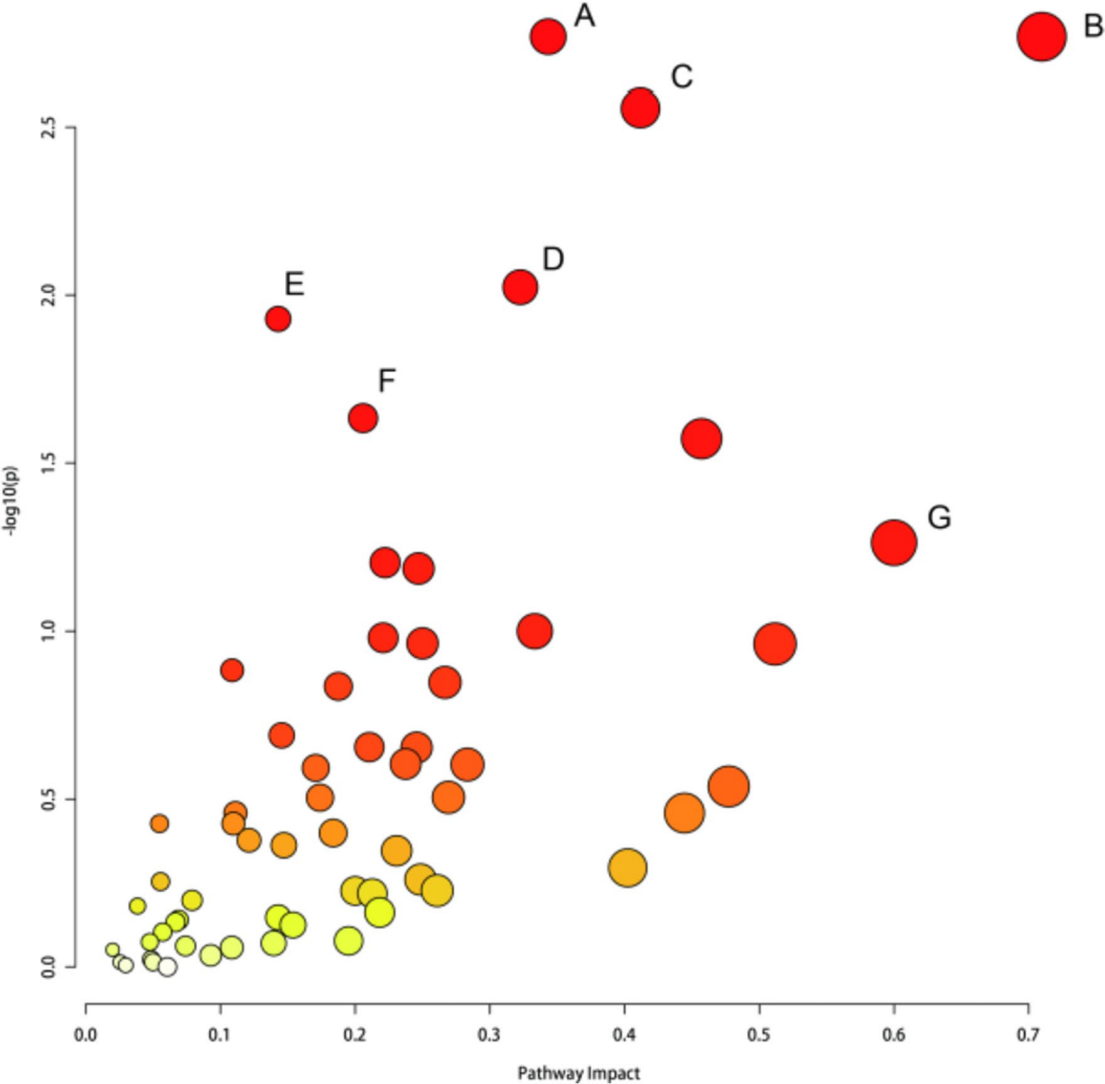
Metabolic pathway analysis of the final differential metabolites and DEGs. **A** Glycine, serine, and threonine metabolism; **B** pentose and glucuronate interconversions; **C** glycerolipid metabolism; **D** histidine metabolism; **E** mucin-type O-glycan biosynthesis; **F** inositol phosphate metabolism; **G** cysteine and methionine metabolism

The biological pathway analysis of metabolite-gene interactions was performed on 106 differential metabolites and 1050 DEGs using the joint pathway analysis module on MetaboAnalyst 5.0 (https://genap.metaboanalyst.ca/MetaboAnalyst/upload/JointUploadView.xhtml). Enrichment analysis showed that the identified proteins and metabolites were significantly enriched in specific pathways (Fig. 7) (*p* < 0.05), including glycine, serine and threonine metabolism, pentose and glucuronate interconversions, glycerolipid metabolism, histidine metabolism, mucin-type o-glycan biosynthesis, inositol phosphate metabolism, and cysteine and methionine metabolism. These seven metabolic pathways may be the key pathways for curcumin to treat osteoarthritis.

**Fig. 8:**
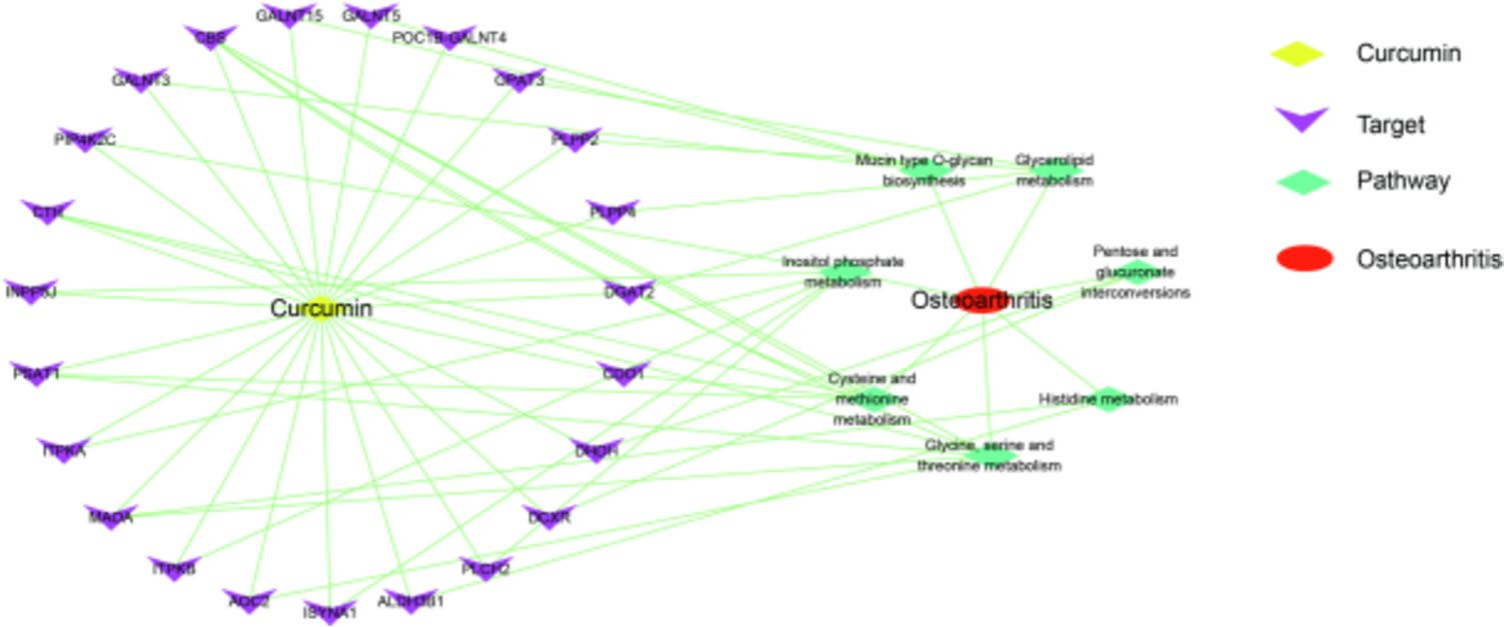
Network of “curcumin-key targets-key pathways-osteoarthritis”

By screening the targets in these seven key pathways, we obtained 23 core targets after deduplication. Detailed information about these targets can be found in Table 2. A network of “curcumin-key target-key pathway-osteoarthritis” was constructed to reveal the mechanism of curcumin in treating osteoarthritis (Fig. 8). In this network, it can be seen that curcumin may exert treatment effects on osteoarthritis via multiple targets and multiple pathways. For instance, curcumin may exhibit therapeutic effects on osteoarthritis by modulating the CBS, CTH, PSAT1, MAOA, and AOC2 proteins involved in the metabolism of glycine, serine, and threonine. Additionally, curcumin can exert therapeutic effects on osteoarthritis by targeting the PIP4K2C, INPP5J, ITPKA, ITPKB, ISYNA1, and PLCH2 proteins associated with inositol phosphate metabolism. Moreover, curcumin may have therapeutic effects on osteoarthritis by interacting with the CTH, CBS, CDO1, and PSAT1 proteins involved in cysteine and methionine metabolism.

**Table 2:** The key targets of curcumin in the treatment of osteoarthritis

No	Target	Uniprot id	Protein name
1	CBS	P35520	Cystathionine beta-synthase
2	ALDH3B1	P43353	Aldehyde dehydrogenase family 3 member B1
3	AOC2	O75106	Retina-specific copper amine oxidase
4	CTH	P32929	Cystathionine gamma-lyase
5	MAOA	P21397	Amine oxidase [flavin-containing] A
6	PSAT1	Q9Y617	Phosphoserine aminotransferase
7	CDO1	Q16878	Cysteine dioxygenase type 1
8	DCXR	Q7Z4W1	L-xylulose reductase
9	DGAT2	Q96PD7	Diacylglycerol O-acyltransferase 2
10	DHDH	Q9UQ10	Trans-1,2-dihydrobenzene-1,2-diol dehydrogenase
11	GALNT15	Q8N3T1	Polypeptide N-acetylgalactosaminyltransferase 15
12	GALNT3	Q14435	Polypeptide N-acetylgalactosaminyltransferase 3
13	GALNT5	Q7Z7M9	Polypeptide N-acetylgalactosaminyltransferase 5
14	GPAT3	Q53EU6	Glycerol-3-phosphate acyltransferase 3
15	INPP5J	Q15735	Phosphatidylinositol 4,5-bisphosphate 5-phosphatase A
16	ISYNA1	Q9NPH2	Inositol-3-phosphate synthase 1
17	ITPKA	P23677	Inositol-trisphosphate 3-kinase A
18	ITPKB	P27987	Inositol-trisphosphate 3-kinase B
19	PIP4K2C	Q8TBX8	Phosphatidylinositol 5-phosphate 4-kinase type-2 gamma
20	PLCH2	O75038	1-Phosphatidylinositol 4,5-bisphosphate phosphodiesterase eta-2
21	PLPP2	O43688	Phospholipid phosphatase 2
22	PLPP4	Q5VZY2	Phospholipid phosphatase 4
23	POC1B-GALNT4	F8VUJ3	Polypeptide N-acetylgalactosaminyltransferase

## Discussion

The treatment of diseases in traditional Chinese medicine is characterized by its multi-component, multi-target, and multi-pathway approach. Metabolomics allows for the detection of changes in endogenous metabolites, while transcriptomics can reveal patterns of gene expression during physiological development and the occurrence of diseases. The combination of transcriptomics and metabolomics provides a promising strategy to unravel the mechanism by which traditional Chinese medicine treats diseases. In our study, we validated the anti-inflammatory properties of curcumin on osteoarthritis in human articular chondrocytes and identified 106 differential metabolites. Clustering analysis of transcriptomics was conducted on the DEGs in the blank group, model group, and curcumin group. A total of 1050 mRNAs were found to be dysregulated in the model group and subsequently rescued by curcumin treatment. By integrating the potential pathways derived from metabolomics and transcriptomics, we identified seven shared pathways as key pathways, namely, glycine, serine, and threonine metabolism; histidine metabolism; pentose and glucuronate interconversions; glycerolipid metabolism; mucin-type O-glycan biosynthesis; inositol phosphate metabolism; cysteine and methionine metabolism; and beta-alanine metabolism. By screening the proteins within these key pathways, we identified a total of 23 key targets.

Current studies have shown that curcumin, a natural compound, possesses promising anti-osteoarthritis properties (Kotha & Luthria, 2019; Zeng et al., 2022; Nakagawa et al., 2020; Thomas et al., 2021). In a mouse model of posttraumatic osteoarthritis (Zhang et al., 2016), curcumin has been found to slow the progression of osteoarthritis and relieve its symptoms by reducing the levels of MMP-1, MMP-3, MMP-13, ADAMTS5, IL-1β, and TNF-α. Consistently, we found that curcumin significantly decreased the levels of inflammatory markers IL-β, IL-6, and TNF-α in human articular chondrocytes, exerting anti-inflammation effects. Acid-activatable curcumin polymer micelles significantly protect joint structures from arthritis by inhibiting TNF-α and IL-1β (Kang et al., 2020). *Curcuma longa* L. was found to be more effective than a placebo in alleviating knee pain without affecting knee effusion-synovitis or cartilage composition (Srivastava et al., 2016). Research has shown that the intake of curcumin twice daily for a duration of 4 weeks improves PGE2 levels in patients with chronic knee osteoarthritis, similar to naproxen (Heidari-Beni & Moravejolahkami, 2020). Various functions of curcumin act synergistically to exert a therapeutic effect on osteoarthritis. Our study demonstrates that curcumin may alleviate osteoarthritis by acting on key proteins in glycine, serine, and threonine metabolism, inhibiting pyruvate production and regulating glycolysis.

After curcumin intervention, there were changes observed in glycine, serine, and threonine metabolism. Specifically, the expression of 2-phospho-d-glyceric acid increased, while the expression of pyruvic acid (pyruvate) and l-methionine decreased. Moreover, the relative mRNA expression levels of cystathionine-β-synthase (CBS), PSAT1, and MAOA were upregulated, while the relative mRNA expression levels of AOC2 and CTH were downregulated. In pentose and glucuronate interconversions, there was an increase in the expression of 2-phospho-d-glyceric acid, but a decrease in the expression of d-glucuronic acid, d-xylitol, and imidazole-4-acetaldehyde. Additionally, the relative expression of DCXR and DHDH mRNA was downregulated. In glycerolipid metabolism, the expression of 2-phospho-d-glyceric acid increased, and the expression of pyruvic acid decreased following curcumin intervention. The relative mRNA expression of DGAT2 and PLPP2 increased, whereas the relative mRNA expression of GPAT3 and PLPP4 decreased. In histidine metabolism, there was an increase in the expression of d-2,3-dihydroxypropanoic acid and a decrease in the expression of d-xylose and urocanic acid. Furthermore, the relative expression of MAOA and ALDH3B1 mRNA increased. In mucin-type O-glycan biosynthesis, the relative mRNA expression of GALNT5 and GALNT15 increased, while the relative mRNA expression of GALNT3 and POC1B-GALNT4 was downregulated. In inositol phosphate metabolism, the expression of imidazole-4-acetaldehyde decreased. Additionally, the relative mRNA expression of PLCH2 increased, while the relative mRNA expression of PIP4K2C, INPP5J, ITPKA, ITPKB, and ISYNA1 was downregulated. Lastly, in cysteine and methionine metabolism, the expression of methylimidazole acetaldehyde and l-methionine decreased. Conversely, the relative mRNA expression of PSAT1 and CBS increased, while the relative mRNA expression of CTH and CDO1 decreased.

Previous research has demonstrated that curcumin, an anti-inflammatory compound, has a potential therapeutic effect in osteoarthritis (Koroljević et al., 2023). Our research results suggest that curcumin may play a role in the treatment of osteoarthritis by regulating the metabolic processes involving glycine, serine, and threonine. Glycine is the predominant amino acid for mammals and other animals. Glycine is synthesized through inter-organ metabolism primarily involving the liver and kidneys, using serine, threonine, choline, and hydroxyproline as precursor molecules. Glycine plays a significant role in metabolic regulation, antioxidant response, and neurological function. Thus, glycine has been utilized in various applications, including the prevention of tissue damage, enhancement of antioxidant capacity, promotion of protein synthesis and wound healing, and amelioration of metabolic disturbances in disorders such as cancer and inflammatory diseases (Wang et al., 2013a). In glycine, serine, and threonine metabolism, pyruvate serves as the end product of glycolysis. Furthermore, pyruvate acts as a pivotal intersection point for various metabolic pathways involved in ATP production and maintaining homeostasis of carbohydrates, fats, and amino acids (Jeoung et al., 2014; Olenchock & Vander Heiden, 2013). Numerous studies have demonstrated the significance of glycolytic metabolism in inflammation (Cheng et al., 2014; Cheng et al., 2016). Lactate, a byproduct of glycolysis, has been identified as a crucial mediator of inflammatory responses in macrophages (Tan et al., 2015; Wei et al., 2015; Peter et al., 2015). Classically activated macrophages rely on glycolysis and glutamine metabolism to produce substantial amounts of succinate, which in turn promotes inflammation and interleukin-1 production (Tannahill et al., 2013). Inhibition of glycolysis is characterized by a decrease in concentrations of pyruvate and lactate (Tan et al., 2020). In mammals, CBS is involved in the initiation and rate-limiting step of the transsulfuration pathway, responsible for generating endogenous H2S (hydrogen sulfide) through the enzymatic pathway. In the cardiovascular system, endogenous H2S catalyzed by CSE (cystathionine-γ-lyase) can regulate vascular tension, promote angiogenesis, and protect the heart (Li et al., 2011). In the nervous system, CBS is primarily found in astrocytes and neural stem cells, contributing to endothelial function protection through its anti-inflammatory, antioxidant, and angiogenic effects (Wang et al., 2013b). Rutin is found to negatively regulate RhoA/ROCK signaling by promoting the expression of CBS, effectively inhibiting the inflammatory progression of osteoarthritis (Sui et al., 2022). Protein kinase RNA-like ER kinase (PERK) is a key metabolic center for the immunosuppressive function of macrophages. The α-KG is an important cofactor for JMJD3 histone demethylation, and JMJD3-α-KG signaling is associated with M2 activation in macrophages (Vitale et al., 2019). Furthermore, α-KG supplementation can alleviate the osteoarthritic phenotype by modulating mitophagy and oxidative stress, suggesting its potential as a therapeutic target to improve osteoarthritis (Liu et al., 2023). The reduced immunosuppressive M2 properties resulting from PSAT1 and PERK deficiency may be due to reduced histone demethylation and limited α-KG availability. The relationship between PERK and PSAT1-mediated serine biosynthesis opens up possibilities for reprogramming or editing M2 macrophages, which may benefit the treatment of cancer or other inflammatory diseases (Raines et al., 2022).

In the pathophysiological process of osteoarthritis, there is a significant increase in pro-inflammatory and pro-catabolic factors, leading to accelerated catabolism and disruption of the dynamic balance of cell metabolism (Loeser, 2011). In this situation, cells respond by increasing anabolism through processes such as proliferation and protein synthesis to maintain the dynamic balance between anabolism and catabolism. Unlike those in a fully differentiated and quiescent state, chondrocytes in an inflammatory microenvironment undergo adaptive changes in their energy metabolism. This is characterized by a significant increase in glycolysis, while reliance on aerobic metabolism through the mitochondrial tricarboxylic acid cycle is greatly reduced (Mobasheri et al., 2017). Chondrocytes also can sense the concentration of oxygen and glucose in the ECM and respond accordingly by regulating cellular metabolism. This adaptive response allows chondrocytes to rely more on anaerobic glycolysis for energy production during periods of acute nutritional and oxygen stress (Lotz & Loeser, 2012).

In the pathogenesis of osteoarthritis, there is excessive production of nitric oxide by chondrocytes, which significantly impairs mitochondrial function and consequently reduces ATP synthesis. To meet the energy demands of chondrocytes, there is an upregulation of glycolysis to enhance ATP production (Lane et al., 2015). There is a significant increase in glycolysis levels in articular chondrocytes of patients with osteoarthritis (Maneiro et al., 2003). The altered energy metabolism in chondrocytes results in a significant reduction in the amount of pyruvate entering the tricarboxylic acid cycle. Instead, pyruvate in the cytoplasm is converted into lactate, a metabolic end product, through the action of lactate dehydrogenase. This accumulation of lactate in the cytoplasm further lowers the pH value of the already acidic microenvironment, leading to extracellular acidosis (Richardson et al., 2010). Research indicates that patients with osteoarthritis have lower pH levels and lower pH measurements are associated with more severe symptoms and impaired knee function (Lombardi et al., 2022).

Mitochondria are the power centers of cells, which provide energy in the form of ATP for cell activity, differentiation, death, signal regulation, and cell cycle control (Shutt & Shadel, 2010). Mitochondria are also molecular platforms for the integration of multiple innate immune signaling pathways (Monlun et al., 2017). Mitochondrial dysfunction and oxidative stress are important hallmarks of abnormal metabolism in osteoarthritis (Loeser et al., 2016). In the process of degenerative diseases including osteoarthritis, changes in mitochondrial structure, dynamics, and genome stability lead to a decline in mitochondrial respiratory function, excessive synthesis of reactive oxygen species (ROS), and oxidative damage. Compared with healthy individuals, the mitochondrial DNA damage of chondrocytes in osteoarthritis patients was significantly increased, while the repair ability was significantly decreased, increasing the apoptosis rate of chondrocytes (Grishko et al., 2009). The maintenance of mitochondrial membrane potential is important for promoting oxidative phosphorylation and synthesis of ATP. The analysis of mitochondrial electron transport chain activities shows that the mitochondrial membrane potential of chondrocytes as well as the content of complexes II and III in patients with osteoarthritis decreased significantly (Maneiro et al., 2003). Although the majority of ATP in chondrocytes comes from glycolysis rather than oxidative phosphorylation (Mobasheri et al., 2005), mitochondrial ROS help maintain cellular redox balance to promote glycolysis (Martin et al., 2012). The weakening of the energy storage capacity of chondrocytes and the shift of metabolic pathway to glycolysis lead to impaired cellular anabolism, decreased ECM synthesis (Johnson et al., 2000; Cillero-Pastor et al., 2013), and reduced cell viability. The massive synthesis of nitric oxide and ROS induces more mitochondrial DNA damage and inhibition of mitochondrial oxidative phosphorylation (Henrotin et al., 2003; Johnson et al., 2001; Reed et al., 2014). Active lipid peroxidation in articular chondrocytes of osteoarthritis patients leads to accelerated mitochondrial DNA damage in chondrocytes, which acts as a feed-forward loop, thereby affecting chondrocyte telomeric DNA and replicative lifespan, and destroying the integrity of cartilage proteoglycans (Martin & Buckwalter, 2002; Yudoh et al., 2005).

## Conclusion

Overall, we postulate that curcumin might have therapeutic potential in the management of osteoarthritis by targeting CBS, PSAT1, MAOA, and other crucial proteins. However, further experimental verification is required to ascertain the regulatory effect of curcumin on specific targets.

## Data Availability

The data that support the findings of this study are available on request from the corresponding author, Qinghu He, upon reasonable request. The RNA-seq data have been deposited in GEO, under the accession number of GSE243421.
